# Dynamic Inertia Weight Binary Bat Algorithm with Neighborhood Search

**DOI:** 10.1155/2017/3235720

**Published:** 2017-05-28

**Authors:** Xingwang Huang, Xuewen Zeng, Rui Han

**Affiliations:** ^1^National Network New Media Engineering Research Center, Institute of Acoustics, Chinese Academy of Sciences, Beijing 100190, China; ^2^University of Chinese Academy of Sciences, Beijing 100049, China

## Abstract

Binary bat algorithm (BBA) is a binary version of the bat algorithm (BA). It has been proven that BBA is competitive compared to other binary heuristic algorithms. Since the update processes of velocity in the algorithm are consistent with BA, in some cases, this algorithm also faces the premature convergence problem. This paper proposes an improved binary bat algorithm (IBBA) to solve this problem. To evaluate the performance of IBBA, standard benchmark functions and zero-one knapsack problems have been employed. The numeric results obtained by benchmark functions experiment prove that the proposed approach greatly outperforms the original BBA and binary particle swarm optimization (BPSO). Compared with several other heuristic algorithms on zero-one knapsack problems, it also verifies that the proposed algorithm is more able to avoid local minima.

## 1. Introduction

There are many optimization problems with binary search space. And many of them are high dimensional. Thus, it is infeasible to solve them with exhaustive method. So as to optimize these problems, such as unit commitment [1], feature selection [2, 3], task scheduling [4, 5], and 0-1 knapsack problem [6, 7], binary algorithms are proposed to generate binary solutions. For example, Mirjalili et al. [8] adapted the standard continuous BA algorithm to be applied to binary spaces and then BBA combined with *k*-Nearest Neighbor (KNN, *k* = 1) method was used to solve feature selection problem [2]. BBA can provide competitive performance but, in some cases, it may get stuck to local minima. To solve this issue, an improved binary bat algorithm, named IBBA, is proposed. IBBA will carry out a more diversified search process. The main contributions of the paper can be summarized as follows:

1. An improved high-performance binary bat algorithm is proposed for binary problems. Using the neighbor bat and dynamic inertia weight strategy, the proposed approach can be more able to avoid being trapped into local minima.
2. To evaluate its performance, the proposed IBBA and several other algorithms are implemented on benchmark functions and zero-one knapsack problems. The results obtained prove that IBBA outperforms the other algorithms.

The organization of the paper is as follows: a compact overview of BA and BBA is given in Section 2. A literature review on inertia weight strategies is also provided in this section.  Section 3 presents the improved structure of IBBA. The experimental results of benchmark functions and zero-one knapsack problems are demonstrated in Sections 4 and 5, respectively. And the reason that the performance of IBBA is better than other algorithms is given in Section 6. Finally, conclusion is made in Section 7.

## 2. Background 

This section provides a brief overview of BA and BBA. A literature review on inertia weight strategies is also presented.

### 2.1. The Bat Algorithm

Inspired by the echolocation behavior of bats, Yang proposed the bat algorithm [9]. When bats chase preys, they will decrease the loudness and increase the frequency of emitted ultrasonic sound. These characteristics of real bats have been used in developing the BA. These basic steps of BA have been mathematically described as follows [9].

In the BA, each bat has three vectors, including a frequency vector, a velocity vector, and a position vector that are updated at time step *t* as (1), (2), and (3):

(1) Vit+1=Vit+Xit−GbestFi

(2) Xit+1=Xit+Vit+1,

where *Gbest* represents the best position obtained so far and *F*_*i*_ represents the frequency of *i*th bat which is updated as follows:

(3) $$\begin{array}{c} F_{i}=F_{min}+\left(\;F_{max}-F_{min}\;\right)\beta , \end{array}$$

where *β* in the range of [0,1] is a random vector drawn from a uniform distribution. From (1) and (3), it is obvious that different frequencies promote the exploration capability of bats to the optimal solution.

These equations, to a certain extent, can guarantee the exploitation capability of the BA. However, to perform the intensification better, a random walk operation has also been employed as follows:

(4) $$\begin{array}{c} X_{\text{new}}=X_{\text{old}}+\varepsilon {\bar{A}}^{t}, \end{array}$$

where *X*_old_ means one solution selected randomly among the current best solutions, *ε* is a randomly selected number in the range of [−1,1], and $\bar{A}$ indicates the average loudness of all bats at this time step. The pseudocode of BA algorithm is demonstrated in Algorithm 1. Note that rand is a random number uniformly distributed in the range [0,1]. To an extent, BA can be considered as a balanced combination of global and intensive local search. The pulse emission rate (*r*) and loudness (*A*) control the balancing between these two search techniques. As *A* increases, artificial bats tend to perform a diversification rather than intensification. These two parameters mentioned above are updated as follows:

(5) Ait+1=αAitrit+1=ri01−exp⁡−γt,

where *α* and *γ* are constants and *α* has the same meaning of the cooling factor in SA [10]. To guarantee that the artificial bats are moving toward the optimal solutions, both loudness and emission rate are updated when the better solutions are found. For any 0 < *α*,  *γ* < 1,

(6) Ait⟶0,rit⟶ri0,as  t⟶∞.

For simplicity, *α* = *γ* can be used.

### 2.2. Binary Bat Algorithm

The binary bat algorithm (BBA) was proposed by Mirjalili et al. [8] to solve optimization problems with binary search space. The structure of BBA is almost the same as the original BA in which the velocity and frequency are defined in continuous space. BBA makes two changes to the original BA:

- The vector of position is no longer a continuous-valued vector but a bit string.
- The random operation demonstrated by (4) is no longer suitable to binary search space. Instead, a simpler operation is adopted.

The position update equation for BBA changes to

(7) $$\begin{array}{c} x_{i}^{k}\left(\;t+1\;\right)=\left\{\begin{array}{cc} {\left(x_{i}^{k}\left(t\right)\right)}^{-1} & rand\leq f\left(v_{i}^{k}\left(t+1\right)\right)\\ x_{i}^{k}\left(t\right) & rand>f\left(v_{i}^{k}\left(t+1\right)\right), \end{array}\right. \end{array}$$

where

(8) $$\begin{array}{c} f\left(\;v_{i}^{k}\left(\;t\;\right)\;\right)=\left|\;\frac{2}{\pi }\arctan\left(\;\frac{\pi }{2}v_{i}^{k}\left(\;t\;\right)\;\right)\;\right| \end{array}$$

and *x*_*i*_^*k*^(*t*) and *v*_*i*_^*k*^(*t*) indicate the position and velocity of *i*th artificial bat at iteration *t* in *k*th dimension and (*x*_*i*_^*k*^(*t*))^−1^ represents the complement of *x*_*i*_^*k*^(*t*).

The operation demonstrated by (4) for BBA changes to

(9) $$\begin{array}{c} X_{\text{new}}=X_{\text{old}}, \end{array}$$

where *X*_old_ still denotes a solution selected randomly from the current best solutions.

### 2.3. Inertia Weight Strategies

In some heuristic algorithms, especially PSO [11], inertia weight strategy plays an important role in the process of keeping balance between global search and local search process. The inertia weight strategy determines the contribution proportion of a particle's old velocity to its new velocity at the current time step. Shi and Eberhart [12] proposed the concept of inertia weight by adopting constant inertia weight strategy and demonstrated that a large inertia weight enhances the exploration while a small inertia weight enhances the exploitation. Further, various dynamic inertia weight strategies have been proposed which can improve the capabilities of PSO and they can be categorized into three classes: constant and random inertia weight strategies, time-varying inertia weight strategies, and adaptive inertia weight strategies. A compact literature review of inertia weight strategies is presented in subsequent paragraphs.

Eberhart and Shi [13] presented a random inertia weight strategy which was demonstrated to be more suitable for dynamic problems. Khan et al. [14] proposed a modified PSO by introducing a mutation mechanism and using dynamic algorithm parameters. To increase the diversity of the particles, the inertia weight of each particle adopts a random updating formula.

Most of the PSO variants employed time-varying inertia weight strategies in which the value of the inertia weight is adjusted based on the iteration number. In [15], a linear decreasing variant of inertia weight was proposed and was illustrated to be effective in enhancing the fine tuning performance of the PSO. Inspired by the idea of decreasing the inertia weight over time step, a nonlinear decreasing inertia weight strategy was proposed [16]. Gao et al. [17] presented a novel PSO variant which combined chaos mutation operator with the logarithm decreasing inertia weight. Based on the idea of decreasing inertia weight, Chen et al. [18] proposed two natural exponent inertia weight strategies to solve the continuous optimization problems.

Adaptive inertia weight strategies is another research trend of inertia weight strategies which monitor the search situation and adjust the inertia weight value according to one or more feedback parameters. Nickabadi et al. [19] proposed a new adaptive inertia weight approach that employs the success rate of the swarm as the feedback parameter to ascertain the particle's situation in the search space. Zhan et al. [20] presented an adaptive particle swarm optimization (APSO) which provides better search efficiency than classical PSO. Yang et al. [21] used speed factor and aggregation degree factor to adapt the value of inertia weight.

## 3. Improved Binary Bat Algorithm

BA is an algorithm combined with many merits of previous proposed heuristic algorithms, such as PSO [11] and SA [10]. Therefore, it stands for the reason that the update process of velocity and location in the BA has many similarities with PSO. When the velocity update equation (1) is analyzed, it is obvious that this equation consists of two parts. The first item (*V*_*i*_(*t*)) denotes the velocity of population and the second item ((*X*_*i*_(*t*) − *Gbest*)*F*_*i*_) controls the velocity of the *i*th position (*X*_*i*_(*t*)) with guidance of the global best solution (*Gbest*). The first and second items of the equation affect the algorithm so that it performs global and local search, respectively. It has been proven that the first item of (1) may reduce the convergence rate rapidly and the second item of (1) may result in premature convergence problem [22]. To solve this problem, some improved BA algorithms have been proposed recently [22–25]. Due to the fact that the structure of BBA is effectively the same as the original BA, BBA is not good enough at exploration and exploitation, too.

**Figure 1:**
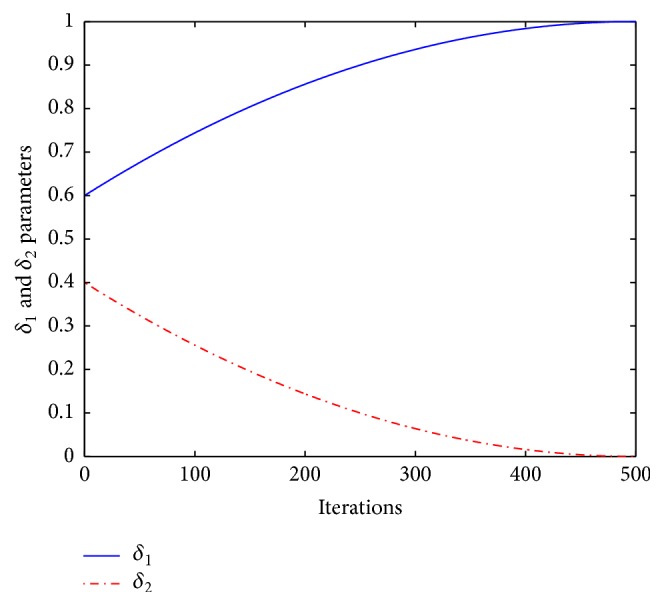
The changes of *δ*_1_ and *δ*_2_ with iterations.

EBA [22] illustrates that the algorithm could produce better solutions with the guidance of the neighbor bat (*k*th solution). For this purpose, inspired by [12], the velocity update equation of original BBA is modified as follows:

(10) Vit+1=wVit+Xit−GbestFiδ1+Xit−XktFiδ2δ1+δ2=1,

where *w* denotes the inertia weight factor which balances local and global search intensity of the *i*th solution by controlling the value of old velocity *V*_*i*_(*t*), *X*_*k*_ represents one of the best solutions randomly selected from the population (*i* ≠ *k*), *δ*_1_ is self-adaptive learning factor of global best solution (*Gbest*) ranging from 0 to 1, and therefore, *δ*_2_, which is a learning factor of *k*th solution, ranges from 1 to 0. Since the *k*th solution information is used to guide the *i*th solution, the algorithm can be more able to avoid local minima. As *δ*_1_ is increased, the effect of the global best solution (*Gbest*) becomes higher than the *k*th neighbor solution (*X*_*k*_) and vice versa. The update equation for *δ*_1_ is shown as follows:

(11) $$\begin{array}{c} \delta_{1}=1+\left(\;\delta_{init}-1\;\right){\left(\;\frac{iter_{max}-iter}{iter_{max}}\;\right)}^{n}, \end{array}$$

where *δ*_init_ denotes initial impact factor of *δ*_1_, iter_max_ represents the maximum number of iterations, iter indicates the current number of iterations, and *n* indicates a nonlinear modulation index. As iter is increased, *δ*_1_ will increase from *δ*_init_ to 1 nonlinearly and *δ*_2_ will decrease from (1 − *δ*_init_) to 0 correspondingly. With a small *δ*_1_ and a large *δ*_2_, bats are allowed to fly around the search space, instead of flying toward the swarm best. On the other hand, a large *δ*_1_ and a small *δ*_2_ allow the bats to converge to the global optimum solution in the latter stages of the search process. Therefore, the proposed approach can effectively control the global search and enhance convergence to the global best solution during the latter part of the optimization. Thus, the solution can switch from exploration to exploitation. The states of *δ*_1_ and *δ*_2_ have been illustrated in Figure 1 when *δ*_init_ and *n* are 0.6 and 2, respectively.

Inspired by [26], dynamic inertia weight strategy is used to control the magnitude of the velocity. This strategy is illustrated as follows:

(12) $$\begin{array}{c} w=w_{max}*\exp\left(\;-m*{\left(\;\frac{iter}{iter_{max}}\;\right)}^{m}\;\right), \end{array}$$

where iter_max_ indicates the total number of iterations, iter denotes the current number of iterations, maximal inertia values are represented by *w*_max_, and *m* is a constant larger than 1.

It is crucial to balance the large-scale exploration search and exploitation search. Once the algorithm locates the approximate position of global optima, refined local exploitation search capabilities need to be enhanced to get global optimum. To dynamically control the transformation point of global search and local search, an adaptive strategy is designed. If the current global best solution is not improved after *q* iterations, the algorithm switches to intensive exploitation search with the smaller *m* or continues exploration search with the current *m*. The strategy is defined as follows:

(13) $$\begin{array}{c} m=\left\{\begin{array}{cc} m & Gbest^{i+q}\geq Gbest^{i}\\ \delta_{1}*\delta_{2}*m & otherwise, \end{array}\right. \end{array}$$

where *Gbest*^*i*+*q*^ and *Gbest*^*i*^ represent the (*i* + *q*)th and *i*th taken values of *Gbest*^*t*^, respectively, and *q* indicates an interval of definite iterations.

According to the descriptions mentioned above, the benefit of the proposed improved binary bat algorithm (IBBA) is that it contributes to the dispersion of the solutions into binary search space. In addition, more accurate results can be obtained.

## 4. Benchmark Experiment

To verify the performance of proposed algorithm, thirteen benchmark functions [27] are employed. These test functions contain two different groups: unimodal and multimodal functions. Tables 1 and 2 demonstrate the benchmark functions used, respectively, where range means the search boundary of the function. The dimensions of all test functions are 5. The global minimum values of all benchmark functions used except *f*_8_(*x*) are 0 while the global minimum value of *f*_8_(*x*) is (−418.9829 × 5).

For this simulation, 15 bits are used to represent each continuous variable in binary. Thus, the dimension of generating bit vector for each benchmark function is calculated as follows:

(14) $$\begin{array}{c} n_{b}=D_{\text{fun}}\times 15, \end{array}$$

where *n*_*b*_ is the dimension of each artificial bat in IBBA and *D*_fun_ represents the dimension of a particular benchmark function. Therefore, the dimensions of the corresponding binary problems are *n*_*b*_ = 75 (5 × 15).

The simulation focused on comparing the performance of the IBBA, BBA [8], and BPSO [28]. Some basic parameters should be initialized before running these algorithms. The initial parameters for these algorithms are demonstrated in Table 3. Tables 4 and 5 present the experimental results. The obtained results of each algorithm are averaged over 30 independent runs, and best results are denoted in bold type. In addition, the mean (Mean), standard deviation value (SD), and medium value (Med) of the optimal solution in the last iteration are presented.

To judge whether IBBA is significantly different from BBA and BPSO or not, we carried out the statistical Wilcoxon's rank-sum test [29] at a 5% significance level. The *p* values calculated in Wilcoxon's rank-sum test comparing IBBA and other algorithms over all the benchmark functions are summarized in Tables 6 and 7. In the demonstrated tables, NA means “Not Applicable” which indicates that the corresponding approach could not statistically compare with itself in the Wilcoxon's rank-sum test. According to Derrac et al. [30], if a *p* value is < 0.05, it can be considered as strong evidence against the null hypothesis. In other words, it means that the compared algorithms have significant difference from each other.

### 4.1. Unimodal Benchmark Functions

Unimodal benchmark functions are effective to quantify the convergence speed. Tables 4 and 6 summarize the results for these benchmark functions. It can be observed from Table 4 that IBBA could get the most optimal values. *p* values in Table 6 also point out that IBBA outperforms the other algorithms significantly. The results obtained prove that the proposed IBBA has excellent performance in finding the global optimal solution of unimodal functions.

**Figure 2:**
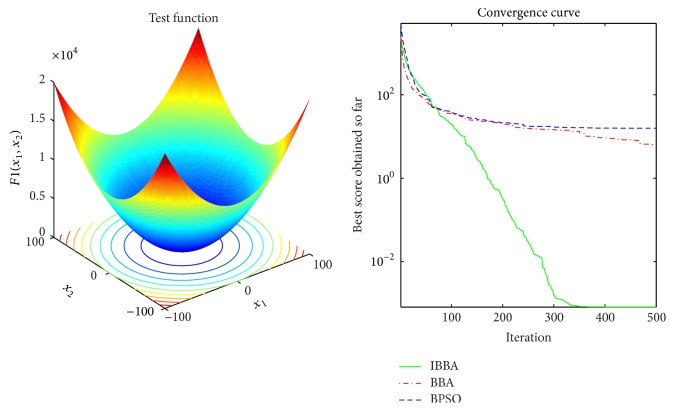
The averaged convergence curve of *f*_1_.

**Figure 3:**
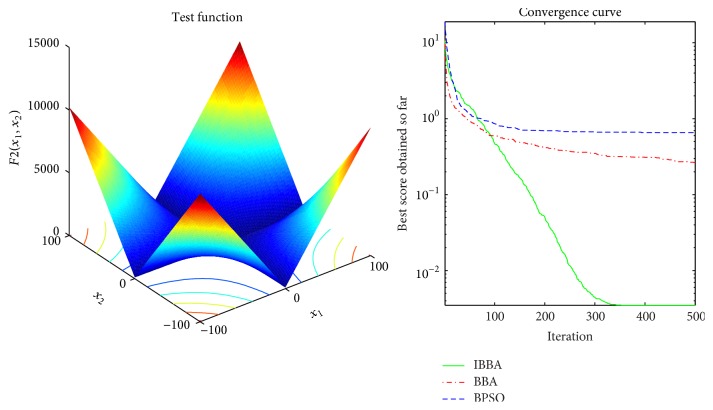
The averaged convergence curve of *f*_2_.

**Figure 4:**
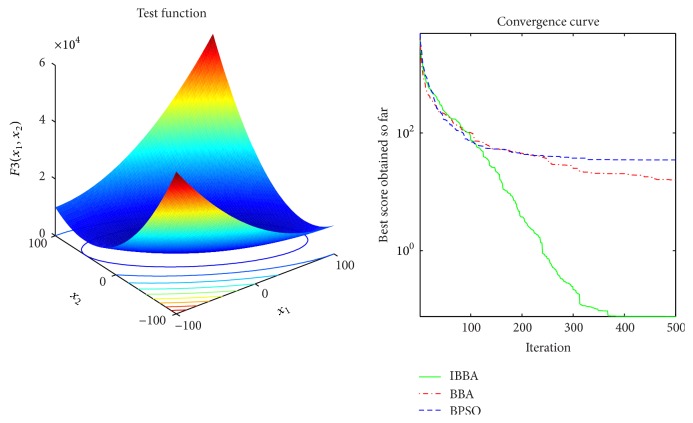
The averaged convergence curve of *f*_3_.

**Figure 5:**
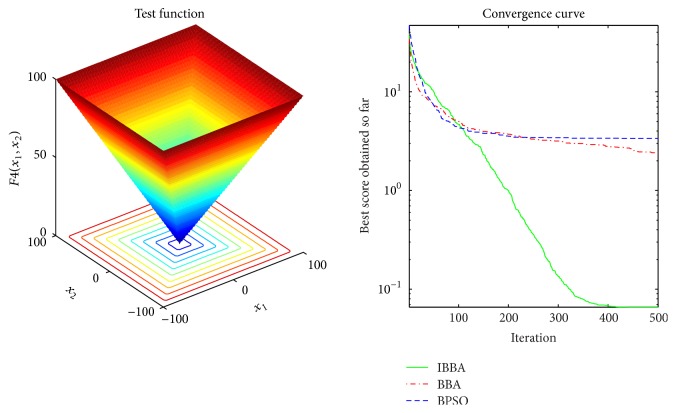
The averaged convergence curve of *f*_4_.

**Figure 6:**
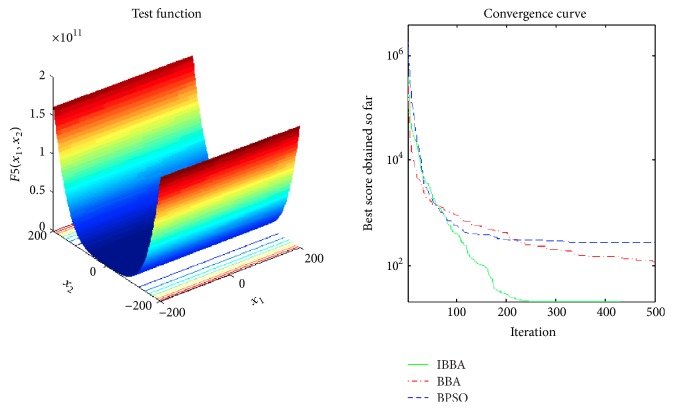
The averaged convergence curve of *f*_5_.

**Figure 7:**
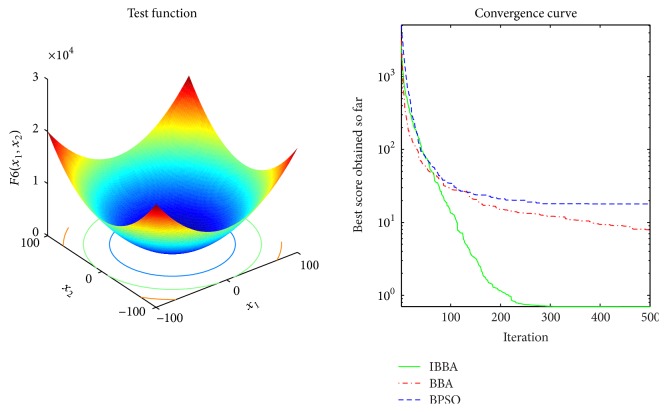
The averaged convergence curve of *f*_6_.

**Figure 8:**
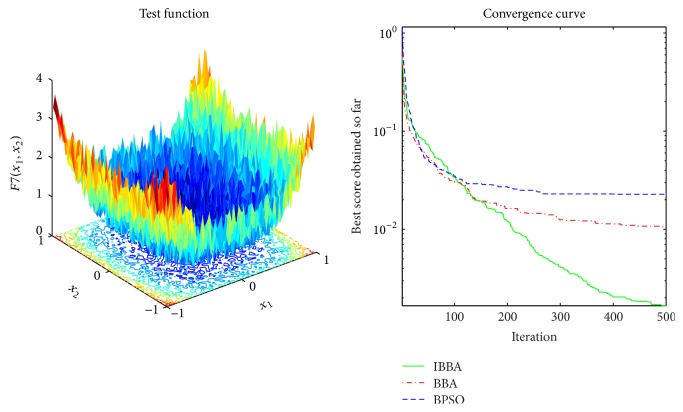
The averaged convergence curve of *f*_7_.

Figures 2–8 illustrate the averaged convergence curves of algorithms on unimodal benchmark functions. As these curves show, the accuracy of the IBBA is much better than other algorithms on these unimodal benchmark functions in terms of the mean, standard deviation value, and median value of the results. According to Tables 4 and 6 and Figures 2–8, it can be concluded that the IBBA is capable of finding the optimal solution with considerable fast convergence rate in unimodal benchmark functions.

### 4.2. Multimodal Benchmark Functions

Multimodal benchmark functions are usually used to detect whether the algorithm faces premature convergence problem. Tables 5 and 7 provide the results of the multimodal benchmark functions.  Table 5 shows that IBBA could get the best results in all cases. And *p* values in Table 7 indicate that, compared to other algorithms, IBBA achieves significant performance boost in all the multimodal benchmark functions. Therefore, this is evidence that IBBA has the best capability of avoiding to be trapped in local minima.

**Figure 9:**
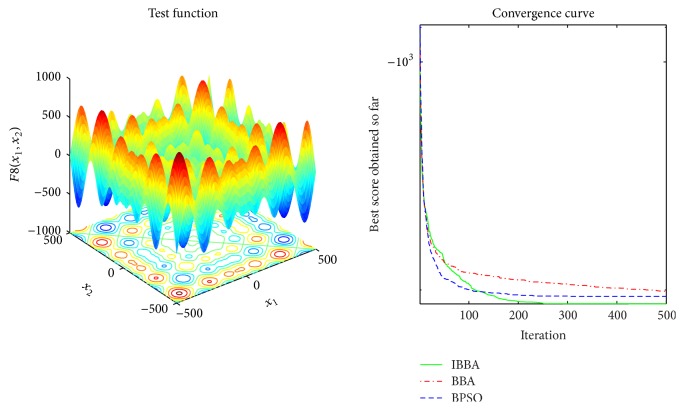
The averaged convergence curve of *f*_8_.

**Figure 10:**
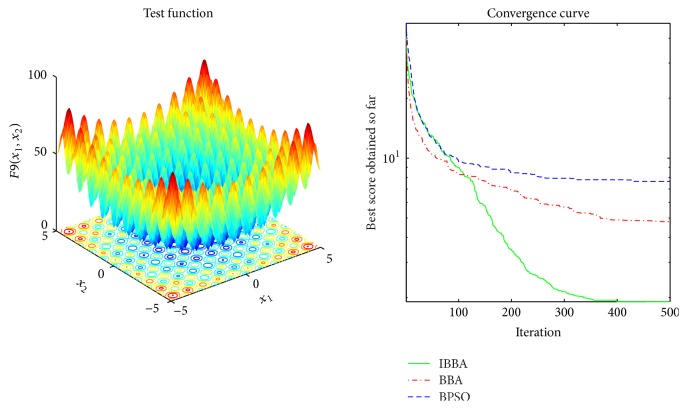
The averaged convergence curve of *f*_9_.

**Figure 11:**
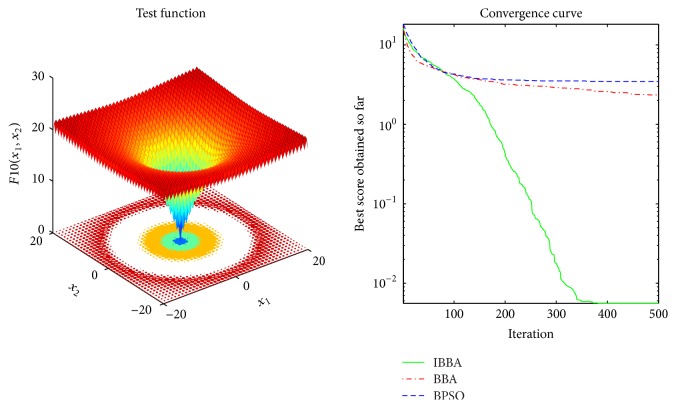
The averaged convergence curve of *f*_10_.

**Figure 12:**
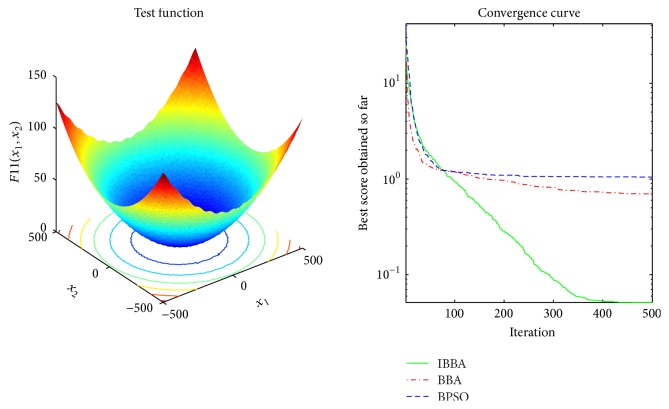
The averaged convergence curve of *f*_11_.

**Figure 13:**
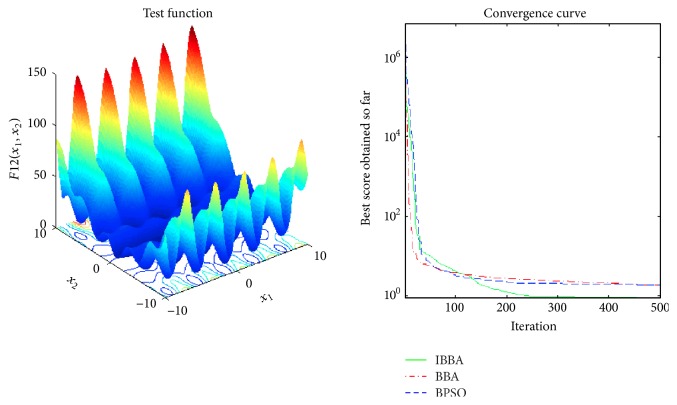
The averaged convergence curve of *f*_12_.

**Figure 14:**
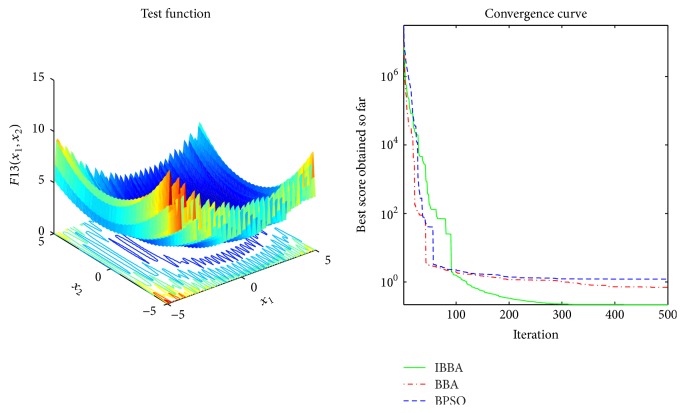
The averaged convergence curve of *f*_13_.

The averaged convergence curves of all algorithms when they deal with multimodal benchmark functions are demonstrated in Figures 9–14. It can be observed from these figures that IBBA also has a significant improvement on the accuracy than other algorithms on multimodal benchmark functions. According to Tables 5 and 7 and Figures 9–14, results obtained prove that the IBBA is able to avoid local minima.

According to the comparison and discussion mentioned above, it can be concluded that the proposed algorithm has huge merit among the binary algorithms. The next section presents the performance of IBBA in solving the zero-one knapsack problems.

## 5. Zero-One Knapsack Problem

Zero-one knapsack problem is a classical combinatorial optimization problem [31] and it has plenty of immediate applications in industry and financial management. It is assumed that, in this problem, there are many items waiting to be carried in knapsack and each item is with a specific benefit and weight. The aim of this problem is to maximize the total profit in the knapsack while the total weight in the knapsack is not more than the given limit. The zero-one knapsack problem can be modeled as follows [31]:

(15) max fx=∑i=1npixis.t. ∑i=1nwixi≤C xi=0,1i=1,2,…,n,

where *n* is the number of items and *x*_1_ to *x*_*n*_ have a benefit *p*_*i*_ and weight *w*_*i*_. The maximum weight that this knapsack can carry is *C*. Assume that all benefits and weights are nonnegative. In this study, five typical knapsack problem datasets are used [7]. These test datasets are listed in Table 8, where Dim represents the dimension of knapsack problems, parameters *w*, *p*, *C* present the information of weight, price, and weight limit, and Opt indicates the optimal value of corresponding knapsack problem. Table 3 is used to set the initial parameters of each algorithm, too. The averaged results obtained are listed in Table 9 and optimal results are denoted in bold type.

According to Table 9, it can be observed that, in all cases, IBBA performs better than the other two algorithms. The maximum results of IBBA in four out of five test datasets are equal to the optimal values of corresponding knapsack problems. In the remaining test datasets (*k*4), the maximum result of IBBA is also almost equal to the optimal values. What is more, the standard deviation values of IBBA are smaller than those of other algorithms, which means that IBBA can search the optimal value more stably. Thus, it could be concluded that IBBA could provide better capability to solve the zero-one knapsack problem than original BBA and BPSO.

## 6. Discussion 

This section briefly presents the reason that the performance of IBBA was better than the other two algorithms:

1. Inspired by several heuristic algorithms, BA combines the merit of previously proposed heuristic algorithms, such as PSO and SA.
2. With the guidance of neighbor bat, the algorithm could produce more diversified solutions.
3. To balance the global search and local search, dynamic inertia weight strategy is employed to control the magnitude of the velocity. Thus, it could avoid premature convergence.
4. The adaptive strategy designed to dynamically control the transformation point of global search and local search promotes the accuracy of results.

## 7. Conclusions 

In this paper, an improved binary bat algorithm is presented. Simple but effective modifications are employed to enhance the BBA. Then the proposed IBBA is implemented and tested on thirteen well-known benchmark functions and three typical zero-one knapsack problems. The obtained results are compared with those of BBA and BPSO. The experiment results show that the proposed approach could get much better performance than the other two algorithms mentioned above in terms of accuracy of the results.

For further studies, we intend to apply the proposed algorithm to practical applications, such as application switch problem [32] and task scheduling problem [4].

## Figures and Tables

**Algorithm 1:**
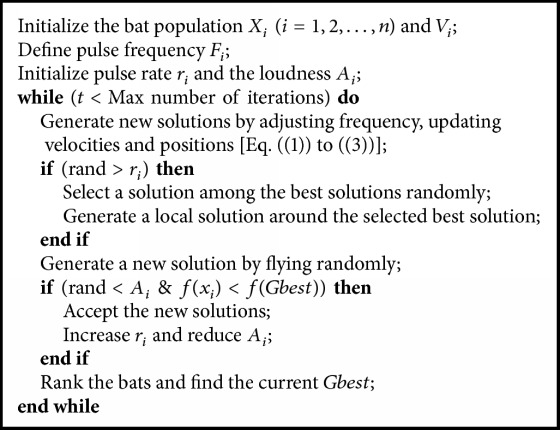
Pseudocode of bat algorithm.

**Table 1:** Unimodal benchmark functions.

Function	Range
$f_{1}(x)=\overset{D}{\underset{i=1}{\sum }}x_{i}^{2}$	[−100,100]
$f_{2}(x)=\overset{D}{\underset{i=1}{\sum }}|x_{i}|+\overset{D}{\underset{i=1}{\prod }}|x_{i}|$	[−10,10]
$f_{3}(x)=\overset{D}{\underset{i=1}{\sum }}{\left(\sum_{j=1}^{i}x_{j}\right)}^{2}$	[−100,100]
$f_{4}(x)=\underset{i}{\max}\left\{|x_{i}|,1\leq i\leq D\right\}$	[−100,100]
$f_{5}(x)=\overset{D-1}{\underset{i=1}{\sum }}\left[100{\left(x_{i+1}-x_{i}^{2}\right)}^{2}+{\left(x_{i}-1\right)}^{2}\right]$	[−30,30]
$f_{6}(x)=\overset{D}{\underset{i=1}{\sum }}{\left(\left\lfloor x_{i}+0.5\right\rfloor \right)}^{2}$	[−100,100]
$f_{7}(x)=\overset{D}{\underset{i=1}{\sum }}ix_{i}^{4}+random[0,1)$	[−1.28,1.28]

**Table 2:** Multimodal benchmark functions.

Function	Range
$f_{8}(x)=\overset{D}{\underset{i=1}{\sum }}-x_{i}\sin\left(\sqrt{\left|x_{i}\right|}\right)$	[−500,500]
$f_{9}\left(x\right)=\overset{D}{\underset{i=1}{\sum }}\left[x_{i}^{2}-10\cos\left(2\pi x_{i}\right)+10\right]$	[−5.12,5.12]
$f_{10}(x)=-20\exp\left(-0.2\sqrt{\frac{1}{D}\overset{D}{\underset{i=1}{\sum }}x_{i}^{2}}\right)-exp\left(\frac{1}{D}\overset{D}{\underset{i=1}{\sum }}cos(2\pi x_{i})\right)$	[−32,32]
$f_{11}(x)=\frac{1}{4000}\overset{D}{\underset{i=1}{\sum }}x_{i}^{2}-\overset{D}{\underset{i=1}{\prod }}\cos\left(\frac{x_{i}}{\sqrt{i}}\right)+1$	[−600,600]
$f_{12}\left(x\right)=\frac{\pi }{D}\left\{10\;\sin^{2}\left(\pi y_{i}\right)+\overset{D-1}{\underset{i=1}{\sum }}{\left(y_{i}-1\right)}^{2}\left[1+10\;\sin^{2}\left(\pi y_{i+1}\right)\right]+{\left(y_{D}-1\right)}^{2}\right\}+\overset{D}{\underset{i=1}{\sum }}u\left(x_{i},10,100,4\right)$	[−50,50]
$y_{i}=1+\frac{1}{4}(x_{i}+1)$	
$u(x_{i},a,k,m)=\left\{\begin{array}{cc} k{\left(x_{i}-a\right)}^{m}, & x_{i}>a,\\ 0, & -a\leq x_{i}\leq a,\\ k{\left(-x_{i}-a\right)}^{m}, & x_{i}<-a, \end{array}\right.$	
$f_{13}(x)=0.1\left\{\sin^{2}\left(3\pi x_{1}\right)+\overset{D-1}{\underset{i=1}{\sum }}{\left(x_{i}-1\right)}^{2}\left[1+\sin^{2}\left(3\pi x_{i}+1\right)\right]+{\left(x_{D}-1\right)}^{2}\left[1+\sin^{2}\left(2\pi x_{D}\right)\right]\right\}+\overset{D}{\underset{i=1}{\sum }}u\left(x_{i},5,100,4\right)$	[−50,50]

**Table 3:** Initial parameters for IBBA, BBA, and BPSO.

Alg.	Parameters	Values
IBBA	Number of bats	30
	*F*_min⁡_	0
	*F*_max⁡_	2
	*A*	0.25
	*r*	0.5
	*ε*	[−1,1]
	*α*	0.9
	*γ*	0.9
	*w*_max⁡_	0.9
	Modulation index, *n*	2
	*m*	50
	*q*	50
	*δ*_init_	0.6
	Max iteration	500
	Stopping criterion	Max iteration

BBA	Number of particles	30
	*F*_min⁡_	0
	*F*_max⁡_	2
	*A*	0.25
	*r*	0.5
	*ε*	[−1,1]
	*α*	0.9
	*γ*	0.9
	Max iteration	500
	Stopping criterion	Max iteration

BPSO	Number of particles	30
	*C*_1_, *C*_2_	2, 2
	*W*	Decreased linearly from 0.9 to 0.4
	Max iterations	500
	Max velocity	6
	Stopping criterion	Max iteration

**Table 4:** Performance comparison on unimodal benchmark functions.

*f*	Metric	IBBA	BBA	BPSO
*f*_1_	Mean	8.38241**e** − 05	5.80234*e* + 00	1.99866*e* + 01
	SD	1.40763**e** − 04	6.36667*e* + 00	1.10249*e* + 01
	Med	4.6569**e** − 05	3.774*e* + 00	1.92831*e* + 01

*f*_2_	Mean	2.95012**e** − 03	3.24087*e* − 01	6.4235*e* − 01
	SD	7.12409**e** − 03	1.59684*e* − 01	2.02785*e* − 01
	Med	1.52593**e** − 03	2.94809*e* − 01	6.5554*e* − 01

*f*_3_	Mean	2.30664**e** − 01	1.3097*e* + 01	2.63465*e* + 01
	SD	9.28325**e** − 01	1.25882*e* + 01	1.72224*e* + 01
	Med	1.39707**e** − 04	8.60949*e* + 00	2.29727*e* + 01

*f*_4_	Mean	4.88296**e** − 03	2.37292*e* + 00	3.5139*e* + 00
	SD	8.94619**e** − 03	1.33978*e* + 00	9.97208*e* − 01
	Med	3.05185**e** − 03	2.03558*e* + 00	3.59813*e* + 00

*f*_5_	Mean	4.38632**e** + 00	1.31671*e* + 02	2.9431*e* + 02
	SD	4.43842**e** + 00	1.50937*e* + 02	2.24461*e* + 02
	Med	3.98383**e** + 00	6.204*e* + 01	2.56548*e* + 02

*f*_6_	Mean	7.05071**e** − 01	6.87477*e* + 00	1.70907*e* + 01
	SD	4.25751**e** − 01	6.73081*e* + 00	1.03668*e* + 01
	Med	7.59196**e** − 01	4.16702*e* + 00	1.6497*e* + 01

*f*_7_	Mean	1.27594**e** − 03	9.92062*e* − 03	2.35084*e* − 02
	SD	9.97156**e** − 04	5.56257*e* − 03	1.30689*e* − 02
	Med	1.23028**e** − 03	9.12516*e* − 03	1.97515*e* − 02

**Table 5:** Performance comparison on multimodal benchmark functions.

*f*	Metric	IBBA	BBA	BPSO
*f*_8_	Mean	−2.08441**e** + 03	−1.99819*e* + 03	−2.03321*e* + 03
	SD	1.83611**e** + 01	7.09513*e* + 01	5.53951*e* + 01
	Med	−2.09464**e** + 03	−2.00568*e* + 03	−2.05689*e* + 03

*f*_9_	Mean	1.30379**e** + 00	4.46724*e* + 00	8.28313*e* + 00
	SD	1.44133**e** + 00	2.75498*e* + 00	1.83015*e* + 00
	Med	1.0004**e** + 00	3.63582*e* + 00	8.39933*e* + 00

*f*_10_	Mean	5.35486**e** − 03	2.35002*e* + 00	3.44122*e* + 00
	SD	5.71445**e** − 03	9.84327*e* − 01	6.31882*e* − 01
	Med	3.95716**e** − 03	2.41859*e* + 00	3.46388*e* + 00

*f*_11_	Mean	4.8303**e** − 02	7.41724*e* − 01⁡	1.05605*e* + 00
	SD	3.35797**e** − 02	2.47447*e* − 01	2.35421*e* − 01
	Med	3.98203**e** − 02	7.20032*e* − 01	1.05621*e* + 00

*f*_12_	Mean	8.11717**e** − 01	2.02809*e* + 00	2.2475*e* + 00
	SD	7.04812**e** − 01	1.89214*e* + 00	1.86339*e* + 00
	Med	6.61436**e** − 01	1.50807*e* + 00	1.63553*e* + 00

*f*_13_	Mean	2.29539**e** − 01	6.26045*e* − 01	1.33228*e* + 00
	SD	1.47623**e** − 01	3.4657*e* − 01	9.26226*e* − 01
	Med	2.54401**e** − 01	5.8134*e* − 01	1.01025*e* + 00

**Table 6:** *p* values on unimodal benchmark functions.

*p* value	IBBA	BBA	BPSO
*f*_1_	N.A.	2.36567*e* − 12	2.36567*e* − 12
*f*_2_	N.A.	4.11097*e* − 12	4.11097*e* − 12
*f*_3_	N.A.	2.58369*e* − 11	2.33455*e* − 11
*f*_4_	N.A.	3.16021*e* − 12	3.16021*e* − 12
*f*_5_	N.A.	1.28366*e* − 09	1.09044*e* − 10
*f*_6_	N.A.	3.65093*e* − 11	2.98783*e* − 11
*f*_7_	N.A.	1.28704*e* − 09	1.61323*e* − 10

**Table 7:** *p* values on multimodal benchmark functions.

*p* value	IBBA	BBA	BPSO
*f*_8_	N.A.	1.35348*e* − 07	6.02212*e* − 07
*f*_9_	N.A.	3.82641*e* − 08	1.04174*e* − 10
*f*_10_	N.A.	1.20954*e* − 11	4.11097*e* − 12
*f*_11_	N.A.	3.68743*e* − 11	3.01797*e* − 11
*f*_12_	N.A.	1.72846*e* − 06	6.00786*e* − 08
*f*_13_	N.A.	8.34855*e* − 08	3.33631*e* − 11

**Table 8:** Five typical test datasets of zero-one knapsack problem.

Number	Dim	Parameters (*w*, *p*, *C*)	Opt
*k*1	10	*w* = (95,4, 60,32,23,72, 80,62, 65, 46), *p* = (55,10,47,5, 4,50,8, 61,85,87), *C* = 269	295

*k*2	20	*w* = (92,4, 43,83,84,68,92,82,6, 44,32,18,56,83,25,96,70,48,14,58), *p* = (44,46,90,72,91,40,75,35,8, 54,78,40,77,15,61,17,75,29,75,63), *C* = 878	1024

*k*3	50	*w* = (80, 82, 85, 70, 72, 70, 66, 50, 55, 25, 50, 55, 40, 48, 59, 32, 22, 60, 30, 32, 40, 38, 35, 32, 25, 28, 30, 22, 50, 30, 45, 30, 60, 50, 20, 65, 20, 25, 30, 10, 20, 25, 15, 10, 10, 10, 4, 4, 2, 1), *p* = (220, 208, 198, 192, 180, 180, 165, 162, 160, 158, 155, 130, 125, 122, 120, 118, 115, 110, 105, 101, 100, 100, 98, 96, 95, 90, 88, 82, 80, 77, 75, 73, 72, 70, 69, 66, 65, 63, 60, 58, 56, 50, 30, 20, 15, 10, 8, 5, 3, 1), *C* = 1000	3103

*k*4	80	*w* = (199, 194, 193, 191, 189, 178, 174, 169, 164, 164, 161, 158, 157, 154, 152, 152, 149, 142, 131, 125, 124, 124, 124, 122, 119, 116, 114, 113, 111, 110, 109, 100, 97, 94, 91, 82, 82, 81, 80, 80, 80, 79, 77, 76, 74, 72, 71, 70, 69, 68, 65, 65, 61, 56, 55, 54, 53, 47, 47, 46, 41, 36, 34, 32, 32, 30, 29, 29, 26, 25, 23, 22, 20, 11, 10, 9, 5, 4, 3, 1), *p* = (40, 27, 5, 21, 51, 16, 42, 18, 52, 28, 57, 34, 44, 43, 52, 55, 53, 42, 47, 56, 57, 44, 16, 2, 12, 9, 40, 23, 56, 3, 39, 16, 54, 36, 52, 5, 53, 48, 23, 47, 41, 49, 22, 42, 10, 16, 53, 58, 40, 1, 43, 56, 40, 32, 44, 35, 37, 45, 52, 56, 40, 2, 23, 49, 50, 26, 11, 35, 32, 34, 58, 6, 52, 26, 31, 23, 4, 52, 53, 19), *C* = 1173	5183

*k*5	100	*w* = (54, 95, 36, 18, 4, 71, 83, 16, 27, 84, 88, 45, 94, 64, 14, 80, 4, 23, 75, 36, 90, 20, 77, 32, 58, 6, 14, 86, 84, 59, 71, 21, 30, 22, 96, 49, 81, 48, 37, 28, 6, 84, 19, 55, 88, 38, 51, 52, 79, 55, 70, 53, 64, 99, 61, 86, 1, 64, 32, 60, 42, 45, 34, 22, 49, 37, 33, 1, 78, 43, 85, 24, 96, 32, 99, 57, 23, 8, 10, 74, 59, 89, 95, 40, 46, 65, 6, 89, 84, 83, 6, 19, 45, 59, 26, 13, 8, 26, 5, 9), *p* = (297, 295, 293, 292, 291, 289, 284, 284, 283, 283, 281, 280, 279, 277, 276, 275, 273, 264, 260, 257, 250, 236, 236, 235, 235, 233, 232, 232, 228, 218, 217, 214, 211, 208, 205, 204, 203, 201, 196, 194, 193, 193, 192, 191, 190, 187, 187, 184, 184, 184, 181, 179, 176, 173, 172, 171, 160, 128, 123, 114, 113, 107, 105, 101, 100, 100, 99, 98, 97, 94, 94, 93, 91, 80, 74, 73, 72, 63, 63, 62, 61, 60, 56, 53, 52, 50, 48, 46, 40, 40, 35, 28, 22, 22, 18, 15, 12, 11, 6, 5), *C* = 3818	15170

**Table 9:** Performance comparison on zero-one knapsack problems over 30 independent runs.

Alg.	Metric	0-1 knapsack problem datasets				
		*k*1	*k*2	*k*3	*k*4	*k*5
IBBA	Avg	**295**	**1024**	**3085.7**	**5134.27**	**15051.6**
	Std	**0**	**0**	**7.32097**	**34.7453**	**35.6122**
	Max	**295**	**1024**	**3103**	**5178**	**15170**
	Min	**295**	**1024**	**3073**	**5063**	**15046**

BBA	Avg	**295**	**1024**	3003.2	4410.67	12942.7
	Std	**0**	**0**	27.3526	95.5894	256.52
	Max	**295**	**1024**	3049	4663	13445
	Min	**295**	**1024**	2944	4251	12489

BPSO	Avg	**295**	959.167	2851.13	3987.77	10089.2
	Std	**0**	23.0563	37.0328	99.9616	238.369
	Max	**295**	1016	2956	4164	10491
	Min	**295**	913	2774	3812	9671
